# Thyroid hormone and intestinal tumor: a Wnt connection

**DOI:** 10.18632/oncotarget.25822

**Published:** 2018-08-10

**Authors:** Yun-Bo Shi

**Affiliations:** Molecular Morphogenesis, National Institute of Child Health and Human Development, National Institutes of Health, Bethesda, MD, USA

**Keywords:** Thyroid hormone receptor, colorectal cancer, adult intestinal stem cells, β-catenin/APC pathway, Wnt

The mammalian intestine has been well-studied as a model system for adult organ-specific stem cells due to the life-long self-renewal of the intestinal epithelium. In the adult, the stem cells near the bottom of the intestinal crypts proliferate and their offspring differentiate into different epithelial cells as they migrate along the crypt-villus axis. The epithelial cells are eventually removed through programmed cell death near the tip of the villus, a process that is conserved in all vertebrates [1-3]. Extensive studies, especially with the use of mouse genetics, have revealed important molecular mechanisms governing intestinal stem cells, including the requirement of the Wnt signaling pathway [1]. Much less is known about the formation of adult intestinal stem cells during vertebrate development. Accumulating evidence indicates that they are formed during the neonatal period in mammals, also known as postembryonic period, when plasma thyroid hormone (T3) levels are high [2, 3]. A role of T3 in the development and function of adult intestinal stem cells is also supported by several other lines of evidence. First, the T3 levels are altered in patients with intestinal abnormalities and diseases [3]. Second, T3 deficiency or knocking out TRα, the predominant TR in mouse intestine, causes intestinal defect, including stem cell proliferation [4]. Finally, human patients with TRα mutations have constipations, suggesting a critical role of TRα in human intestine [5].

Intestinal tumors, particularly, colorectal cancers, affects a large fraction of the human population. These tumors are derived due to dysregulated epithelial cell proliferation. Not surprisingly, the Wnt pathway, which are critical for stem cell development and function [4], are involved in intestinal tumor development and can interact with the β-catenin/APC pathway, which is a critical player in human colorectal cancer [6]. Given the effect of T3 on intestinal stem cell development and/or function, one may expect that T3 signaling affect intestinal tumor development. Indeed, TRβ mutations and changes in TR expression have been reported in intestinal tumors [3]. Furthermore, transgenic overexpression studies have shown that TRα1 enhances tumor development in an APC-mutated mouse model with over-activation of Wnt signaling [7].

A recent study by M. Plateroti and her colleagues provides clinical evidence and molecular insights into the synergy between TRα1 and Wnt signaling pathway in intestinal tumor development [8]. Through bioinformatics analysis of a human colon cancer database, the authors discovered that increased TR expression is correlated with high Wnt activity in colorectal cancer samples. Functional studies in Caco2 cells demonstrated that TRα1 regulates Wnt activity to affect colon cancer cell proliferation and migration. By using their intestinal tumor model with overexpressed TRα1 for transcriptomic analysis, the authors discovered that TRα1 represses the expression of Wnt inhibitors, including Frzb, Sox17 and Wif1. Importantly, bioinformatics analysis of the human colon cancer database showed that these Wnt inhibitors were expressed at lower levels in tumors than in normal tissues and that their expression had an inverted correlation with that of TRα1 in human colorectal cancer patients. These findings not only highlight the importance of increased TRα1 expression in intestinal cancer development and progression but also reveal that Wnt signaling pathway plays a critical role in mediating T3 signaling in intestinal tumorigenesis. They further suggest that like T3 signaling, dysregulation of other factors that participate in the development of adult intestinal stem cells may also contribute to intestinal tumor development and progression.
